# Ethyl 3-acetyl-4-(3-meth­oxy­phen­yl)-6-methyl-2-sulfanyl­idene-1,2,3,4-tetra­hydro­pyrimidine-5-carboxyl­ate

**DOI:** 10.1107/S1600536812017679

**Published:** 2012-04-28

**Authors:** Nikhath Fathima, H Nagarajaiah, Noor Shahina Begum

**Affiliations:** a Department of Studies in Chemistry, Bangalore University, Bangalore 560 001, Karnataka, India

## Abstract

In the title compound, C_17_H_20_N_2_O_4_S, the aryl ring is positioned perpendicular to the dihydro­pyrimidine ring, the dihedral angle between the ring planes being 77.48 (9)°. The carboxyl­ate and methyl groups are in a *cis* conformation with respect to the C=C bond. The dihydro­pyrimidine ring adopts a twist-boat conformation. The crystal structure is stabilized by N—H⋯O and C—H⋯O inter­actions, the former resulting in mol­ecular chains along the *b* axis and the latter forming inversion dimers.

## Related literature

For the biological activity of dihydro­pyrimidines, see: Kappe (2000 ▶). For a related structure, see: Begum & Vasundhara (2009 ▶).
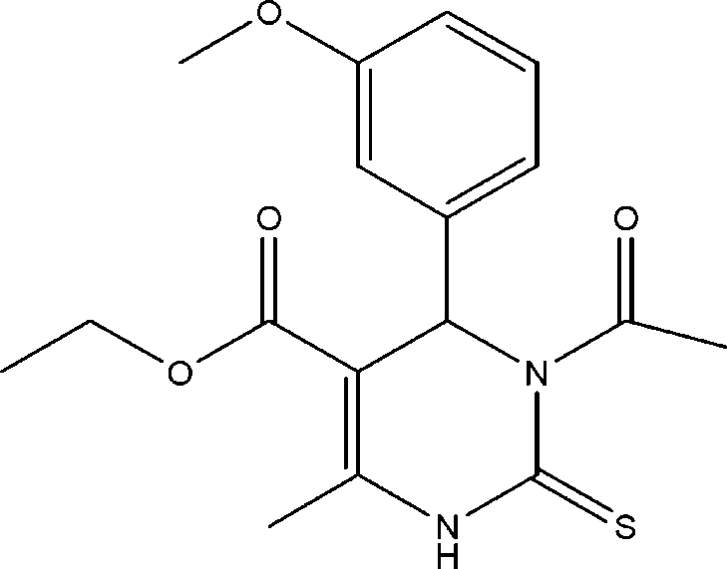


## Experimental

### 

#### Crystal data

C_17_H_20_N_2_O_4_S
*M* *_r_* = 348.41
Monoclinic, 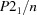

*a* = 11.515 (2) Å
*b* = 7.3687 (16) Å
*c* = 20.049 (4) Å
β = 90.960 (4)°
*V* = 1701.0 (6) Å^3^
*Z* = 4
Mo *K*α radiation
μ = 0.21 mm^−1^
*T* = 296 K
0.18 × 0.16 × 0.16 mm

#### Data collection

Bruker SMART APEX CCD detector diffractometer
Absorption correction: multi-scan (*SADABS*; Bruker, 1998 ▶) *T*_min_ = 0.963, *T*_max_ = 0.967
10054 measured reflections
3707 independent reflections
2545 reflections with *I* > 2σ(*I*)
*R*_int_ = 0.042

#### Refinement

*R*[*F* ^2^ > 2σ(*F* ^2^)] = 0.063
*wR*(*F* ^2^) = 0.204
*S* = 1.03
3707 reflections
221 parameters
H-atom parameters constrained
Δρ_max_ = 0.34 e Å^−3^
Δρ_min_ = −0.43 e Å^−3^

### 

Data collection: *SMART* (Bruker, 1998 ▶); cell refinement: *SAINT-Plus* (Bruker, 1998 ▶); data reduction: *SAINT-Plus*; program(s) used to solve structure: *SHELXS97* (Sheldrick, 2008 ▶); program(s) used to refine structure: *SHELXL97* (Sheldrick, 2008 ▶); molecular graphics: *ORTEP-3* (Farrugia, 1997 ▶) and *CAMERON* (Watkin *et al.*, 1996 ▶); software used to prepare material for publication: *WinGX* (Farrugia, 1999 ▶).

## Supplementary Material

Crystal structure: contains datablock(s) global, I. DOI: 10.1107/S1600536812017679/pv2529sup1.cif

Structure factors: contains datablock(s) I. DOI: 10.1107/S1600536812017679/pv2529Isup2.hkl

Supplementary material file. DOI: 10.1107/S1600536812017679/pv2529Isup3.cml

Additional supplementary materials:  crystallographic information; 3D view; checkCIF report

## Figures and Tables

**Table 1:** Hydrogen-bond geometry (Å, °)

*D*—H⋯*A*	*D*—H	H⋯*A*	*D*⋯*A*	*D*—H⋯*A*
N2—H2⋯O3^i^	0.86	2.07	2.907 (3)	164
C3—H3*B*⋯O1^ii^	0.96	2.60	3.393 (4)	140

Symmetry codes: (i) 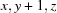; (ii) 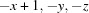.

**Fig. 1.**
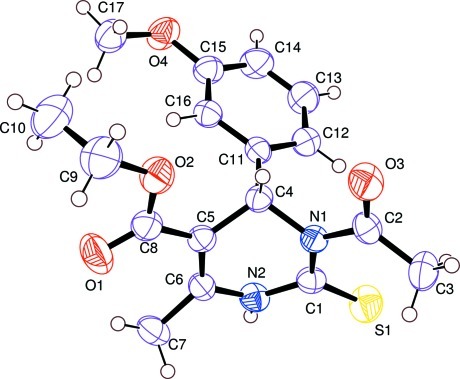
The molecular structure of the title compound with the atom numbering scheme. Displacement ellipsoids are drawn at the 50% probability level. H atoms are presented as small spheres of arbitrary radius.

**Fig. 2.**
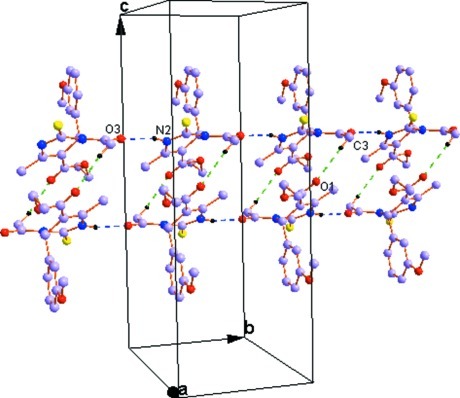
A view of the intermolecular hydrogen bonds (dotted lines) in the crystal structure of the title compound. H atoms non participating in H-bonding were ommitted for clarity.

## supplementary crystallographic information

### Comment

Dihydropyrimidines have remarkable potency with antiviral, antitumor, antibacterial and anti-inflammatory activities, and are used as antihypertensive agents and calcium channel modulators (Kappe, 2000). In the structure of the title compound, the aryl ring substituted to chiral carbon atom (C4) is positioned axially to the dihydropyrimidine ring, whereas the carboxylate, methyl, acetyl and the thioxo groups are attached on the either sides of the ring. The dihedral angle between the planes of the aryl and dihydropyrimidine rings is 77.48 (9)°. The exocyclic ester at C5 adopts a *cis* orientation with respect to C5═C6 double bond. The central dihydropyrimidine ring is significantly puckered, assuming a conformation of twisted boat with the atoms N1 and C1 displaced from the mean plane of the remaining ring atoms (C4/C5/C6/N2) by 0.806 (4) Å and 0.517 (4) Å, respectively. The crystal structure is stabilized by intermolecular interactions N—H···O resulting in molecular chains along the crystallographic *b*-axis. The structure is further consolidated by intermolecular C—H···O interactions resulting in the formation of centrosymmetric dimers about inversion centers (Fig 2.) For a crystal structure related to the title compound, see: Begum & Vasundhara (2009).

### Experimental

4-(3-Methoxy-phenyl)-6-methyl-2-thioxo-1,2,3,4-tetrahydro-pyrimidine-5 carboxylic acid ethyl ester (3.06 g, 10 mmol) was mixed with acetic anhydride (20 ml) and refluxed for about 4 h. The reaction mixture was cooled and diluted by addition of water (20 ml). The solid separated, washed with water, filtered and dried (Yield: 1.92 g, 85% and mp 430-432 K). Pale yellow crystals of the title compound were obtained for diffraction by slow evaporation from a solution in chloroform.

### Refinement

The H atoms were placed at calculated positions in the riding model approximation with N—H = 0.86Å, C—H = 0.93, 0.96, 0.97 and 0.98 Å for aryl, methyl, methylene and methyne H-atoms respectively, with *U*_iso_(H) = 1.5*U*_eq_(C) for methyl H atoms and *U*_iso_(H) = 1.2*U*_eq_(N/C).

### Crystal data

**Table:**

C_17_H_20_N_2_O_4_S	*F*(000) = 736
*M**_r_* = 348.41	*D*_x_ = 1.360 Mg m^−^^3^
Monoclinic, *P*2_1_/*n*	Mo *K*α radiation, λ = 0.71073 Å
Hall symbol: -P 2yn	Cell parameters from 3707 reflections
*a* = 11.515 (2) Å	θ = 2.0–27.0°
*b* = 7.3687 (16) Å	µ = 0.21 mm^−^^1^
*c* = 20.049 (4) Å	*T* = 296 K
β = 90.960 (4)°	Block, yellow
*V* = 1701.0 (6) Å^3^	0.18 × 0.16 × 0.16 mm
*Z* = 4	

### Data collection

**Table:**

Bruker SMART APEX CCD detector diffractometer	3707 independent reflections
Radiation source: fine-focus sealed tube	2545 reflections with *I* > 2σ(*I*)
Graphite monochromator	*R*_int_ = 0.042
ω scans	θ_max_ = 27.0°, θ_min_ = 2.0°
Absorption correction: multi-scan (*SADABS*; Bruker, 1998)	*h* = −12→14
*T*_min_ = 0.963, *T*_max_ = 0.967	*k* = −9→9
10054 measured reflections	*l* = −23→25

### Refinement

**Table:**

Refinement on *F*^2^	Primary atom site location: structure-invariant direct methods
Least-squares matrix: full	Secondary atom site location: difference Fourier map
*R*[*F*^2^ > 2σ(*F*^2^)] = 0.063	Hydrogen site location: inferred from neighbouring sites
*wR*(*F*^2^) = 0.204	H-atom parameters constrained
*S* = 1.03	*w* = 1/[σ^2^(*F*_o_^2^) + (0.1146*P*)^2^ + 0.3834*P*] where *P* = (*F*_o_^2^ + 2*F*_c_^2^)/3
3707 reflections	(Δ/σ)_max_ < 0.001
221 parameters	Δρ_max_ = 0.34 e Å^−^^3^
0 restraints	Δρ_min_ = −0.43 e Å^−^^3^

### Special details

**Table:**

Geometry. All s.u.'s (except the s.u. in the dihedral angle between two l.s. planes) are estimated using the full covariance matrix. The cell s.u.'s are taken into account individually in the estimation of s.u.'s in distances, angles and torsion angles; correlations between s.u.'s in cell parameters are only used when they are defined by crystal symmetry. An approximate (isotropic) treatment of cell s.u.'s is used for estimating s.u.'s involving l.s. planes.
Refinement. Refinement of *F*^2^ against ALL reflections. The weighted *R*-factor *wR* and goodness of fit *S* are based on *F*^2^, conventional *R*-factors *R* are based on *F*, with *F* set to zero for negative *F*^2^. The threshold expression of *F*^2^ > 2σ(*F*^2^) is used only for calculating *R*-factors(gt) *etc*. and is not relevant to the choice of reflections for refinement. *R*-factors based on *F*^2^ are statistically about twice as large as those based on *F*, and *R*-factors based on ALL data will be even larger.

### Fractional atomic coordinates and isotropic or equivalent isotropic displacement parameters (Å^2^)

**Table:**

	*x*	*y*	*z*	*U*_iso_*/*U*_eq_	
C1	0.6452 (2)	0.0344 (3)	0.14742 (12)	0.0409 (6)	
C2	0.6628 (2)	−0.3005 (3)	0.13502 (13)	0.0425 (6)	
C3	0.7912 (2)	−0.2995 (4)	0.12735 (17)	0.0603 (8)	
H3A	0.8280	−0.2751	0.1697	0.090*	
H3B	0.8123	−0.2072	0.0960	0.090*	
H3C	0.8162	−0.4157	0.1113	0.090*	
C4	0.4706 (2)	−0.1548 (3)	0.13863 (12)	0.0362 (5)	
H4	0.4507	−0.2765	0.1224	0.043*	
C5	0.4207 (2)	−0.0197 (3)	0.08906 (11)	0.0377 (5)	
C6	0.4725 (2)	0.1430 (3)	0.08596 (12)	0.0392 (6)	
C7	0.4401 (3)	0.3040 (4)	0.04509 (14)	0.0525 (7)	
H7A	0.3746	0.2748	0.0169	0.079*	
H7B	0.5045	0.3383	0.0180	0.079*	
H7C	0.4204	0.4029	0.0740	0.079*	
C8	0.3207 (2)	−0.0652 (4)	0.04555 (13)	0.0485 (7)	
C9	0.1922 (3)	−0.2962 (5)	0.00900 (18)	0.0734 (10)	
H9A	0.1954	−0.4267	0.0031	0.088*	
H9B	0.1998	−0.2402	−0.0345	0.088*	
C10	0.0772 (3)	−0.2451 (7)	0.0375 (2)	0.0975 (14)	
H10A	0.0713	−0.2945	0.0817	0.146*	
H10B	0.0158	−0.2929	0.0098	0.146*	
H10C	0.0709	−0.1153	0.0395	0.146*	
C11	0.4234 (2)	−0.1375 (3)	0.20943 (12)	0.0380 (5)	
C12	0.4961 (2)	−0.1400 (4)	0.26504 (13)	0.0481 (6)	
H12	0.5763	−0.1422	0.2603	0.058*	
C13	0.4480 (3)	−0.1390 (4)	0.32820 (14)	0.0586 (8)	
H13	0.4968	−0.1423	0.3656	0.070*	
C14	0.3303 (3)	−0.1334 (4)	0.33644 (14)	0.0575 (8)	
H14	0.2998	−0.1332	0.3791	0.069*	
C15	0.2568 (2)	−0.1281 (4)	0.28122 (14)	0.0480 (6)	
C16	0.3032 (2)	−0.1295 (3)	0.21776 (13)	0.0428 (6)	
H16	0.2540	−0.1251	0.1805	0.051*	
C17	0.0614 (3)	−0.1270 (5)	0.23868 (15)	0.0603 (8)	
H17A	0.0741	−0.0239	0.2105	0.090*	
H17B	−0.0168	−0.1248	0.2544	0.090*	
H17C	0.0737	−0.2365	0.2138	0.090*	
O1	0.2712 (2)	0.0368 (4)	0.00752 (12)	0.0801 (8)	
O2	0.28828 (17)	−0.2390 (3)	0.05194 (10)	0.0567 (5)	
O3	0.60926 (17)	−0.4423 (3)	0.13563 (11)	0.0606 (6)	
O4	0.14055 (17)	−0.1211 (3)	0.29420 (10)	0.0607 (6)	
N1	0.59906 (16)	−0.1391 (3)	0.13862 (10)	0.0368 (5)	
N2	0.57304 (19)	0.1684 (3)	0.12438 (11)	0.0434 (5)	
H2	0.5913	0.2783	0.1345	0.052*	
S1	0.76922 (7)	0.08775 (12)	0.18297 (5)	0.0651 (3)	

### Atomic displacement parameters (Å^2^)

**Table:**

	*U*^11^	*U*^22^	*U*^33^	*U*^12^	*U*^13^	*U*^23^
C1	0.0441 (13)	0.0361 (13)	0.0426 (13)	−0.0028 (10)	0.0040 (10)	−0.0003 (10)
C2	0.0491 (14)	0.0370 (14)	0.0413 (13)	0.0064 (11)	−0.0005 (11)	−0.0020 (10)
C3	0.0470 (16)	0.0604 (19)	0.073 (2)	0.0127 (14)	−0.0017 (14)	−0.0011 (15)
C4	0.0344 (12)	0.0333 (12)	0.0408 (13)	−0.0016 (9)	0.0001 (10)	−0.0020 (9)
C5	0.0386 (12)	0.0397 (13)	0.0349 (12)	0.0014 (10)	0.0043 (10)	−0.0005 (10)
C6	0.0426 (13)	0.0398 (14)	0.0353 (12)	0.0066 (10)	0.0037 (10)	0.0016 (10)
C7	0.0661 (18)	0.0433 (15)	0.0484 (16)	0.0069 (13)	0.0059 (13)	0.0105 (12)
C8	0.0436 (14)	0.0605 (18)	0.0414 (14)	−0.0028 (13)	0.0010 (11)	0.0043 (12)
C9	0.073 (2)	0.080 (2)	0.066 (2)	−0.0215 (18)	−0.0174 (17)	−0.0050 (18)
C10	0.059 (2)	0.134 (4)	0.099 (3)	−0.022 (2)	−0.010 (2)	0.016 (3)
C11	0.0412 (13)	0.0338 (13)	0.0391 (13)	0.0014 (10)	−0.0008 (10)	0.0037 (10)
C12	0.0457 (14)	0.0522 (16)	0.0464 (15)	−0.0006 (12)	−0.0009 (11)	0.0042 (12)
C13	0.0581 (18)	0.077 (2)	0.0406 (15)	−0.0021 (15)	−0.0058 (12)	0.0020 (14)
C14	0.0617 (18)	0.072 (2)	0.0388 (15)	−0.0040 (15)	0.0076 (13)	−0.0009 (13)
C15	0.0484 (15)	0.0483 (16)	0.0476 (15)	0.0005 (12)	0.0083 (12)	0.0007 (12)
C16	0.0445 (14)	0.0444 (15)	0.0395 (13)	0.0005 (11)	0.0018 (11)	0.0033 (11)
C17	0.0472 (16)	0.075 (2)	0.0592 (18)	0.0029 (14)	0.0061 (13)	0.0026 (15)
O1	0.0780 (16)	0.0834 (17)	0.0776 (16)	−0.0116 (13)	−0.0360 (13)	0.0318 (13)
O2	0.0535 (12)	0.0591 (13)	0.0570 (12)	−0.0108 (9)	−0.0138 (9)	−0.0023 (9)
O3	0.0610 (13)	0.0308 (10)	0.0900 (17)	0.0025 (9)	−0.0015 (11)	−0.0033 (9)
O4	0.0478 (11)	0.0860 (16)	0.0487 (12)	−0.0003 (10)	0.0123 (9)	−0.0003 (10)
N1	0.0379 (11)	0.0323 (11)	0.0403 (11)	−0.0005 (8)	0.0005 (8)	−0.0009 (8)
N2	0.0506 (12)	0.0293 (11)	0.0504 (13)	−0.0006 (9)	0.0024 (10)	−0.0021 (9)
S1	0.0572 (5)	0.0578 (5)	0.0797 (6)	−0.0096 (4)	−0.0160 (4)	−0.0046 (4)

### Geometric parameters (Å, º)

**Table:**

C1—N2	1.366 (3)	C9—C10	1.500 (6)
C1—N1	1.395 (3)	C9—H9A	0.9700
C1—S1	1.633 (3)	C9—H9B	0.9700
C2—O3	1.213 (3)	C10—H10A	0.9600
C2—N1	1.400 (3)	C10—H10B	0.9600
C2—C3	1.489 (4)	C10—H10C	0.9600
C3—H3A	0.9600	C11—C12	1.383 (4)
C3—H3B	0.9600	C11—C16	1.398 (3)
C3—H3C	0.9600	C12—C13	1.391 (4)
C4—N1	1.484 (3)	C12—H12	0.9300
C4—C5	1.513 (3)	C13—C14	1.369 (4)
C4—C11	1.534 (3)	C13—H13	0.9300
C4—H4	0.9800	C14—C15	1.383 (4)
C5—C6	1.341 (3)	C14—H14	0.9300
C5—C8	1.471 (3)	C15—O4	1.369 (3)
C6—N2	1.393 (3)	C15—C16	1.388 (4)
C6—C7	1.486 (4)	C16—H16	0.9300
C7—H7A	0.9600	C17—O4	1.427 (4)
C7—H7B	0.9600	C17—H17A	0.9600
C7—H7C	0.9600	C17—H17B	0.9600
C8—O1	1.207 (3)	C17—H17C	0.9600
C8—O2	1.341 (4)	N2—H2	0.8600
C9—O2	1.453 (4)		
			
N2—C1—N1	113.0 (2)	C9—C10—H10A	109.5
N2—C1—S1	119.74 (19)	C9—C10—H10B	109.5
N1—C1—S1	127.20 (19)	H10A—C10—H10B	109.5
O3—C2—N1	117.6 (2)	C9—C10—H10C	109.5
O3—C2—C3	120.7 (2)	H10A—C10—H10C	109.5
N1—C2—C3	121.6 (2)	H10B—C10—H10C	109.5
C2—C3—H3A	109.5	C12—C11—C16	119.4 (2)
C2—C3—H3B	109.5	C12—C11—C4	121.7 (2)
H3A—C3—H3B	109.5	C16—C11—C4	118.7 (2)
C2—C3—H3C	109.5	C11—C12—C13	119.3 (3)
H3A—C3—H3C	109.5	C11—C12—H12	120.4
H3B—C3—H3C	109.5	C13—C12—H12	120.4
N1—C4—C5	108.44 (18)	C14—C13—C12	121.4 (3)
N1—C4—C11	111.24 (19)	C14—C13—H13	119.3
C5—C4—C11	114.62 (19)	C12—C13—H13	119.3
N1—C4—H4	107.4	C13—C14—C15	119.9 (3)
C5—C4—H4	107.4	C13—C14—H14	120.1
C11—C4—H4	107.4	C15—C14—H14	120.1
C6—C5—C8	121.3 (2)	O4—C15—C14	115.9 (2)
C6—C5—C4	117.0 (2)	O4—C15—C16	124.6 (3)
C8—C5—C4	121.6 (2)	C14—C15—C16	119.6 (3)
C5—C6—N2	117.5 (2)	C15—C16—C11	120.5 (2)
C5—C6—C7	129.2 (2)	C15—C16—H16	119.8
N2—C6—C7	113.3 (2)	C11—C16—H16	119.8
C6—C7—H7A	109.5	O4—C17—H17A	109.5
C6—C7—H7B	109.5	O4—C17—H17B	109.5
H7A—C7—H7B	109.5	H17A—C17—H17B	109.5
C6—C7—H7C	109.5	O4—C17—H17C	109.5
H7A—C7—H7C	109.5	H17A—C17—H17C	109.5
H7B—C7—H7C	109.5	H17B—C17—H17C	109.5
O1—C8—O2	121.7 (3)	C8—O2—C9	115.5 (2)
O1—C8—C5	126.1 (3)	C15—O4—C17	117.6 (2)
O2—C8—C5	112.2 (2)	C1—N1—C2	125.9 (2)
O2—C9—C10	111.7 (3)	C1—N1—C4	116.71 (19)
O2—C9—H9A	109.3	C2—N1—C4	117.22 (19)
C10—C9—H9A	109.3	C1—N2—C6	125.8 (2)
O2—C9—H9B	109.3	C1—N2—H2	117.1
C10—C9—H9B	109.3	C6—N2—H2	117.1
H9A—C9—H9B	107.9		
			
N1—C4—C5—C6	−38.9 (3)	C12—C11—C16—C15	−1.4 (4)
C11—C4—C5—C6	86.0 (3)	C4—C11—C16—C15	175.0 (2)
N1—C4—C5—C8	140.3 (2)	O1—C8—O2—C9	1.5 (4)
C11—C4—C5—C8	−94.7 (3)	C5—C8—O2—C9	−178.2 (2)
C8—C5—C6—N2	−174.1 (2)	C10—C9—O2—C8	−82.3 (4)
C4—C5—C6—N2	5.2 (3)	C14—C15—O4—C17	−176.1 (2)
C8—C5—C6—C7	3.9 (4)	C16—C15—O4—C17	4.1 (4)
C4—C5—C6—C7	−176.8 (2)	N2—C1—N1—C2	156.5 (2)
C6—C5—C8—O1	−5.4 (4)	S1—C1—N1—C2	−25.5 (4)
C4—C5—C8—O1	175.4 (3)	N2—C1—N1—C4	−28.8 (3)
C6—C5—C8—O2	174.3 (2)	S1—C1—N1—C4	149.21 (19)
C4—C5—C8—O2	−4.9 (3)	O3—C2—N1—C1	171.0 (2)
N1—C4—C11—C12	−10.5 (3)	C3—C2—N1—C1	−12.0 (4)
C5—C4—C11—C12	−134.0 (2)	O3—C2—N1—C4	−3.7 (3)
N1—C4—C11—C16	173.2 (2)	C3—C2—N1—C4	173.4 (2)
C5—C4—C11—C16	49.7 (3)	C5—C4—N1—C1	52.0 (3)
C16—C11—C12—C13	1.6 (4)	C11—C4—N1—C1	−75.0 (2)
C4—C11—C12—C13	−174.7 (3)	C5—C4—N1—C2	−132.9 (2)
C11—C12—C13—C14	−0.8 (5)	C11—C4—N1—C2	100.2 (2)
C12—C13—C14—C15	−0.2 (5)	N1—C1—N2—C6	−10.4 (3)
C13—C14—C15—O4	−179.5 (3)	S1—C1—N2—C6	171.47 (19)
C13—C14—C15—C16	0.4 (4)	C5—C6—N2—C1	22.8 (4)
O4—C15—C16—C11	−179.8 (2)	C7—C6—N2—C1	−155.5 (2)
C14—C15—C16—C11	0.4 (4)		

### Hydrogen-bond geometry (Å, º)

**Table:**

*D*—H···*A*	*D*—H	H···*A*	*D*···*A*	*D*—H···*A*
N2—H2···O3^i^	0.86	2.07	2.907 (3)	164
C3—H3*B*···O1^ii^	0.96	2.60	3.393 (4)	140

Symmetry codes: (i) *x*, *y*+1, *z*; (ii) −*x*+1, −*y*, −*z*.
